# Exploring Tactile Stimuli from a Wrist-Worn Device to Manipulate Subjective Time Based on the Filled-Duration Illusion †

**DOI:** 10.3390/s22197194

**Published:** 2022-09-22

**Authors:** Kiichi Shirai, Kyosuke Futami, Kazuya Murao

**Affiliations:** 1 Graduate School of Information Science and Engineering, Ritsumeikan University, 1-1-1 Nojihigashi, Kusatsu, Shiga 525-8577, Japan; 2 Digital Spirits Teck, Kusatsu 525-8577, Japan

**Keywords:** tactile stimulus, filled-duration illusion, information presentation, perception of time passing, psychological effect, waiting time, mind, mindless computer, design implications

## Abstract

There are situations where manipulating subjective time would be desirable, such as reducing waiting time, and there are many studies to manipulate subjective time. However, it is not easy to use previous methods in various situations because most of them use visual and auditory information. This study proposes a method to manipulate the subjective time by the tactile stimuli from wrist-worn devices. We designed three types of tactile stimuli presentation methods that change the number, the duration, and the time interval of the stimuli. The evaluation result clarified the elements of the tactile stimuli that intentionally changed the subjective time and confirmed that our method can change the subjective time by about 23% (from −6% to +17%). Since few studies have focused on the phenomenon in which the subjective time changes depending on the tactile stimuli from information devices, our findings can contribute to designing information devices and user experiences.

## 1. Introduction

Since people cannot manipulate subjective time, manipulating subjective time would be desirable in various situations. Subjective time means the length of time that people perceive subjectively, and manipulating subjective time means manipulating phenomena that cause subjective time to feel longer or shorter. One such situation is waiting time using a computer. Even though Internet data transfer and computer processing are continuously improving, waiting time is still a problem when installing software, downloading files, completing tasks on a computer, etc. [1]. In fact, it has been investigated that such waiting time for web services can lead to changes in user behavior [2,3,4,5,6,7].

There are many studies that manipulate subjective time to reduce waiting time. Firstly, there are visual information presentation methods, such as a method that moves visual objects on a head-mounted display (HMD) [8] and a method that presents a progress bar [2,3,9,10]. Secondly, there are auditory information presentation methods, such as a method that changes the time interval and frequency of auditory stimuli [1]. The previous studies have demonstrated that perceptual stimuli from information devices can manipulate the subjective time.

However, it is difficult to use the previous methods “always and at any time” in various daily life situations. There are several reasons. Firstly, auditory and visual information presentation is undesirable for some situations. For example, visual and auditory information should not be used for manipulating the subjective time if that information is used for any other purpose. In addition, the visual and auditory channels should be kept free when the eyes and ears need to be used to perceive situations and objects. Secondly, wearing an information presentation device of visual and sound information at any time is difficult for some situations. For example, wearing earphones at all times is difficult for some people because it blocks the ear canal. In addition, wearing HMDs at all times is difficult because of the burden of wearing them.

Therefore, manipulating subjective time using perceptual channels other than visual and auditory channels would be useful in any situation in which visual or auditory information presentation is difficult to use. On the other hand, wearable devices that can present tactile vibration have become popular, such as smartphones and smartwatches. If tactile stimuli can manipulate subjective time, the situations where subjective time can be manipulated can be expanded.

In addition, it is a problem that few studies have focused on clarifying the relationship between tactile stimuli and subjective time even though the number of devices that present tactile stimuli has increased. If there is a phenomenon in which tactile stimuli affect subjective time, information devices designed without considering this phenomenon may cause problems that change the subjective time in a way that impairs the user’s experience. Therefore, clarifying both the existence and the manipulation method of the phenomenon in which tactile stimuli from information devices change the subjective time is useful for preventing such problems and realizing information devices and applications that take into account the effects of tactile stimuli on the subjective time.

**Figure 1:**
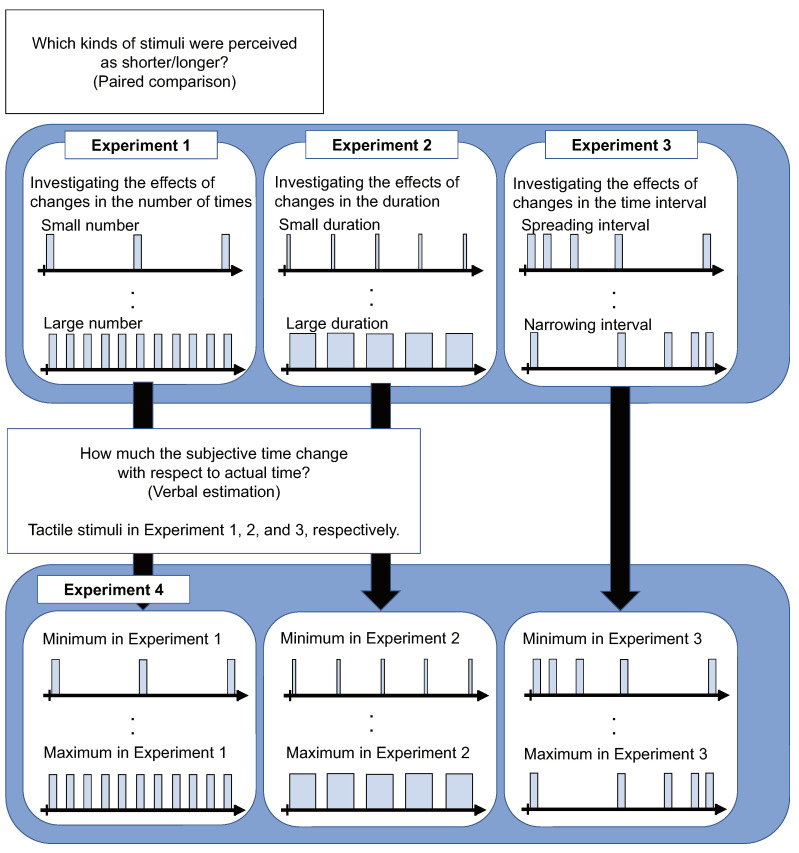
Overview of our study.

This study proposes a method for manipulating subjective time using wrist-worn devices’ tactile stimuli. The purpose of this study is to clarify how subjective time changes depending on the type of tactile stimulus presentation of wearable devices. We created a wrist-worn device that displays tactile stimuli and designed three types of tactile stimuli presentation methods that change the number of stimuli, the duration of stimuli, and the time interval of stimuli. We performed the four experiments shown in Figure 1. In Experiments 1, 2, and 3, we investigated whether our tactile stimuli presentation methods change the subjective time to be perceived as longer or shorter. We prepared three kinds of patterns for each tactile stimuli presentation method. Then, subjects selected the one they felt was longer than the other by comparing two kinds of patterns. In Experiment 4, we investigated how much the subjective time was changed by the tactile stimuli with respect to the objective time. We selected six stimulus patterns from Experiments 1, 2, and 3. Then, subjects answered the estimated length of the subjective time during the perception of each stimulus pattern. From the results, we clarified the elements of the tactile stimuli that changed the subjective time and confirmed that the proposed method can change the subjective time by about 23% (from −6% to +17%).

While most previous methods used auditory and visual stimuli, our method uses tactile stimuli. With our method, users can manipulate the subjective time in the situations mentioned above where visual and auditory information should not be used for manipulating the subjective time. Wrist-worn devices can present tactile stimuli in any situation. Our method can be applied to any information devices such as mobile, wearable, and ubiquitous devices. Our study contributes to the field of human–computer interaction and wearable computing.

Note that this paper extends the previous paper [11]. Although the previous paper showed the possibility that the subjective time is changed by changing the number of tactile stimuli, this paper increased the types of experiments to investigate the factor of the tactile stimuli to change the subjective time and increased the number of subjects to show more reliable results. Specifically, we added the following points: Experiment 1 is the same as the previous paper, examining the effect of the number of stimuli on the subjective time. However, the number of subjects was increased by 50%, which provides more reliable results than the previous paper. Experiment 2 examines the effect of the duration of the stimuli on the subjective time. Experiment 3 examines the effect of the time interval of the stimuli on the subjective time. Experiment 4 examines the degree to which the three factors (i.e., the number, the duration, and the time interval of the stimuli) change the subjective time with respect to the objective time. In addition, this paper added a discussion based on these experiments.

## 2. Related Research

### 2.1. Techniques for Manipulating the Subjective Time

The subjective time in our study refers to the length of time one feels for a certain experience. There are situations in which people’s experience becomes good or not by the subjective time. One of the examples is the waiting time during the use of computers, such as loading time. Previous studies have investigated that user behavior [12,13] and the impression of the service [2,3,4,5,6,7] are changed by the waiting time.

On the other hand, psychological findings show that perceptual stimuli can unconsciously cause subjective changes in time. One of the most famous phenomena is the filled-duration illusion (FDI) [14,15]. This is a phenomenon in which the more information there is in a given amount of time, the longer the subjective time feels. For example, the subjective time with a faster speed stimulus is longer than that with a slower speed stimulus. In fields such as psychology, it has been reported that this phenomenon can be caused by visual stimuli [16], auditory stimuli [14,17,18], and tactile stimuli [14]. However, the results of these studies were merely the discovery of a phenomenon, not a proposal and realization of subjective time control technology.

Based on these studies, many studies have been conducted to manipulate this phenomenon using information devices. Firstly, there are many methods that use visual information. A method has been proposed to shorten the subjective time by providing visual feedback of task progress with a progress bar [2,3,9,10]. This approach can be described as presenting the current processing status of the computer directly to the user. Furthermore, methods of manipulating the progress bar and adding elements have been proposed. For example, there are methods to add animations such as blinking to the progress bar [19,20], a method to add interactive elements to the progress bar [21], a method to change the shape and progress of the progress bar [22], and methods to animate the progress bar to move backward while decelerating [19,20]. Shimizu et al. [8] realized a method to manipulate the subjective time at any time by changing the moving speed of the visual stimuli in the HMD.

Secondly, a method to manipulate the subjective time using auditory information of information devices instead of visual information has also been proposed. Komatsu et al. [1] proposed a method to change the number and time interval of auditory stimuli to reduce the waiting time in computer task processing. There is also a method to add sounds to the progress bar to present the current processing state [23] and a method to use characteristic sounds [24,25,26].

These previous studies showed that the subjective time can be manipulated by stimuli from information devices. In addition, by implementing a method that uses different perceptual channels, such as visual and auditory stimuli, they demonstrated that the usage scenes of subjective time manipulation methods could be expanded. In our study, we employed tactile stimuli and a wrist-worn device that can be worn at all times. Our study enables the use of the subjective time manipulation method in situations where it is difficult to use sound and visual information. Furthermore, our findings can be useful for designing information devices and applications that take into account the effects of tactile stimuli on the subjective time.

### 2.2. Techniques for Manipulating Illusions, Psychological Effects, and Cognitive Biases

Our study supports the user by manipulating the effects that tactile stimuli have on the subjective time. Similar to our study, many methods support users by creating unconscious phenomena (e.g., illusions, psychological effects, and cognitive biases) by using information devices.

In recent years, the technology that uses unconscious phenomena to unconsciously change the user’s body, mind, and behavior is also called mindless computing. Such unconscious phenomena are caused by an automatic mind in a dual-process theory. An automatic mind [27,28] causes emotional and unreasonable reactions, such as cognitive bias, illusions, and psychological effects. This is because an automatic mind is an area where the person’s consciousness does not reach and works according to information perception automatically, at high speed, and with very low energy. Although a reflected mind [27,29] is an area that is controlled by the person’s consciousness, a reflected mind cannot prevent the unconscious phenomena caused by an automatic mind. Because of this characteristic, it is possible to cause desirable reactions in users by intentionally manipulating unconscious phenomena with information devices.

There are methods to improve users’ mental and physical functions. Costa et al. proposed a method to present a different heart rate than the actual one to improve user’s cognitive performance and decrease user’s anxiety level [30,31,32]. These methods employed tactile stimuli from wearable devices so that they can be used constantly in daily life. To improve users’ mental functions, Futami et al. [33] proposed a method to present auditory stimuli and Tagami et al. [34] proposed a method to present a pseudo-success experience in VR space. Costa et al. [35] proposed a method to modulate one’s own voice and others’ voices to reduce the psychological stress caused by voice perception. To improve work efficiency, Ban et al. [36] proposed a method that visually changes the speed of the clock and Kim et al. [37] proposed a method to present a work productivity log based on framing effects. Di et al. [38] investigated and clarified the psychological effects caused by the different timelines that show the remaining time on a task. Arakawa et al. proposed a method to increase the user’s concentration on the video materials for learning by modulating the video’s audio depending on the loss of the user’s concentration, which is detected with machine learning [39]. Michael et al. proposed a method to improve the motivation of cycling training by presenting an environment to compete with ghosts having the user’s future skill and ghosts having the user’s past skills [40].

In addition, there are methods to change experience, sensation, and behaviors. Many methods to manipulate subjective time have been proposed as mentioned above. Ban et al. [41] proposed a method to manipulate subjective fatigue by changing the color of an object to be lifted using VR technology. Futami et al. proposed a method to manipulate load perception by using the psychological phenomenon caused by observing myoelectric sensor information [42]. Wuertz et al. proposed a method to enhance enjoyment and change behavior during a game by presenting the game’s fitness gauge as less than it actually is [43]. Takeuchi et al. [44] showed that others’ evaluations (e.g., likes) of user’s meal contents affect user’s meal choices and taste preferences, then they proposed a method to make users choose a healthy diet by presenting others’ evaluations that were different from the actual ones. Futami et al. proposed a method to manipulate walking motivation caused by competition progress information of step count logs [45] and a method to induce the behavior of not missing the train by showing the train timetable in an ingenious way [46]. Suzuki et al. [47] proposed a method to improve the output of collaborative work over online video communication by changing the facial expressions of dialogue partners from the actual ones.

Humans cannot change their mental and physical states, behaviors, and sensations easily by themselves. For this problem, previous studies showed that it is possible for users to unconsciously manipulate their bodies and minds by using illusions and psychological effects. In addition, these previous studies have shown that clarifying what happens to users due to the use of information devices is important for spreading information devices. Similar to these previous studies, our study investigates the illusion caused by perceptual stimuli from information devices and develops a method to support users by utilizing the illusion.

The necessity of studies that focus on the discovery of the existence and mitigation methods of cognitive biases that are caused by the use of information systems has been emphasized [48]. In our study, we clarify how tactile stimuli from information devices affect the subjective time. There is little knowledge about how tactile stimuli technology affects the user’s experience [49]. Therefore, our study can be useful to improve and design the user’s experience in the use of information devices that can present tactile stimuli.

## 3. Method

In this section, we describe the method of manipulating the subjective time using tactile stimuli from wearable devices, the hypothesis of our study, and the created system.

We adopted a wrist-worn device as the wearable device. Wrist-worn devices are widely used as smartwatches and activity meters. The wrist-worn devices can present stimuli to the user while the user is performing tasks or activities. A previous study [32] also adopted a tactile stimulus on a wrist-worn device so that users could always experience the illusion caused by heart rate information throughout their daily lives. Therefore, it is a suitable device for this research, aiming to manipulate subjective time using tactile stimuli in daily life. Although there are head-worn devices such as smart glasses that are currently in practical use, we did not adopt them because it is not common to wear them all the time at present.

### 3.1. Hypothesis to Be Verified

In this study, we investigated the following points:

- Verification 1: This paper verifies whether the perception of tactile stimuli from information devices causes the phenomenon of changing the subjective time. In addition, this paper verifies the feasibility of a method to manipulate the phenomenon. For example, if the subjective time changes with a certain trend in response to a specific pattern of tactile stimuli, the subjective time can be manipulated intentionally. If this method is revealed, it is possible to intentionally design the effects of the tactile stimuli on the subjective time. It is also possible to design information devices and applications that take into account the effects of tactile stimuli on the subjective time. However, if this phenomenon is caused by a haphazard law, the manipulation of this phenomenon is impossible.
- Verification 2: This paper verifies the degree of the change in the subjective time that is caused by the tactile stimuli. For example, the previous study [1] reported that a method that changes the subjective time by 7.6% over 10 s is useful in actual usage scenarios. It is unclear whether this level of change in the subjective time can be caused by tactile stimuli.

### 3.2. Design of the Stimuli

**Figure 2:**
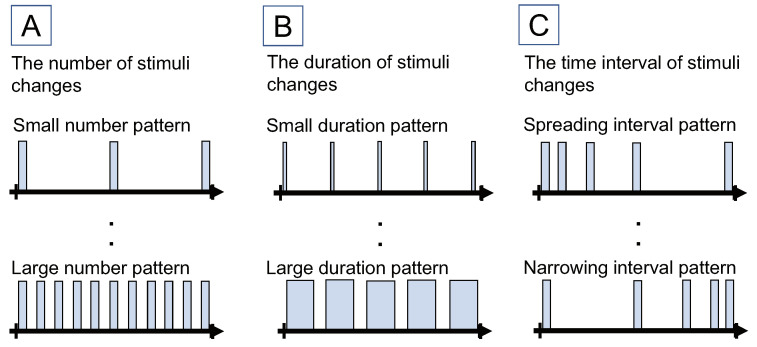
Proposed methods of presenting tactile stimuli. (**A**) is a method that changes the number of the stimuli. (**B**) is a method that changes the duration of the stimuli. (**C**) is a method that changes the time interval of the stimuli. Reprinted/adapted with permission from Ref. [11] 2021, ACM.

We designed the following three methods of presenting the tactile stimuli. Each method varies the number, duration, and time interval of the tactile stimuli. All tactile stimuli were of recognizable intensity and painless to the user. Each element is shown in Figure 2:

- **Method for changing the number of stimuli**
  This method manipulates the number of stimuli. The number of stimuli is shown on the left in Figure 2. In this study, we assumed that the subjective time becomes longer or shorter when the number of stimuli increases or decreases, respectively. This hypothesis is based on the following reasons. First, previous studies of the filled-duration illusion have reported that the subjective time changes depending on the amount of stimuli satisfying a certain time range [14,15]. Secondly, in the previous studies on the manipulation of the subjective time, there are examples of changing the subjective time by changing the amount of visual stimuli [8] and auditory stimuli [1]. However, the tendency of the change in the subjective time was different between visual stimuli and auditory stimuli. For this, the effect of tactile stimulation is unclear.
- **Method for changing the duration of stimuli**
  This method manipulates the duration of the stimuli. The duration of the stimuli is shown in the center of Figure 2. In this study, we assumed that the subjective time becomes longer or shorter when the duration of the stimuli increases or decreases. This hypothesis is also based on the filled-duration illusion.
- **Method for changing the time interval of stimuli**
  This method manipulates the time interval of the stimuli. The time interval of the stimuli is shown in the right part in Figure 2. In this study, we assumed that the subjective time becomes longer or shorter when the time interval gradually increases or decreases. This hypothesis is based on the following reasons. First, the previous study of the manipulation of the subjective time using auditory stimuli [1] changed the same factors of the time interval of the stimuli. Second, previous studies on the effects of visual progress bars on the subjective time have reported that the acceleration and deceleration patterns of the stimuli affect the subjective time [19,20].

## 4. Implementation

**Figure 3:**
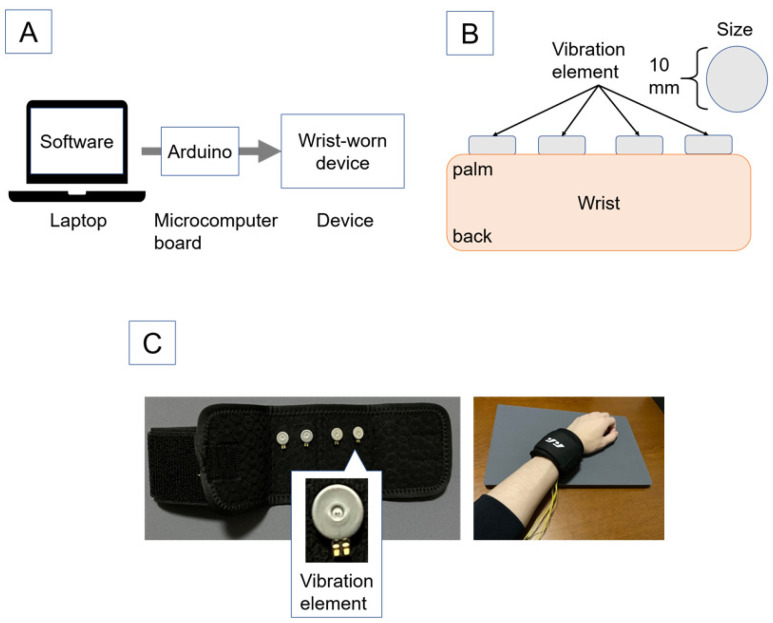
System configuration and implementation device. (**A**) is the system configuration. (**B**) is a diagram of the implementation device. (**C**) is the implemented wrist-worn device. Reprinted/adapted with permission from Ref. [11] 2021, ACM.

We implemented a prototype system of the proposed method. The entire prototype system consists of a wrist-worn device that presents the tactile stimuli, a microcomputer board (Arduino), a laptop, and software for presenting the stimuli. The system configuration is shown in Figure 3. The wrist-worn device that presents the tactile stimuli is shown in Figure 3. The vibrator for the tactile stimuli was a disk-shaped brushless vibration motor (Nidec Copal Electronics Inc., LBV10B-009). In order to prevent the subjects from not being able to feel the stimuli due to their weak tactile sensitivity, we attached four vibration stimuli. The software was implemented using Arduino and Processing. Specifically, we implemented the system using Arduino for creating and presenting the tactile stimulus patterns and Processing for sending the stimulus commands. Since it is necessary to inform subjects of the start point and end point of the stimuli during the experiment, these points were presented on the computer screen by sound and text.

## 5. Experiment 1

In this experiment, we evaluated whether a change in the subjective time occurred in response to the change in the number of tactile stimuli. The number of subjects was 30 (24 males and 6 females), and the mean age was 23.7 years (from 20 to 52 years).

This experiment evaluated the change in the subjective time in 10 s. This reason is as follows. The first reason is that 10 s was used in the previous study using an auditory stimulus [1]. By experimenting with the same time range as the previous study, we can compare this study with the previous study regarding the degree of the change in the subjective time. The second reason is that finding stimulus patterns that make the subjective time of 10 s feel shorter than the objective time of 10 s is reported as useful [1] based on the fact that a waiting time over 10 s changes the user in several points (e.g., task concentration [50], experience satisfaction [3], withdrawal rate of Internet video viewers [51]). The third reason is to investigate the effects of the tactile stimuli with a longer time than the previous study using the tactile stimuli of 1 s [14].

### 5.1. Stimuli

**Figure 4:**
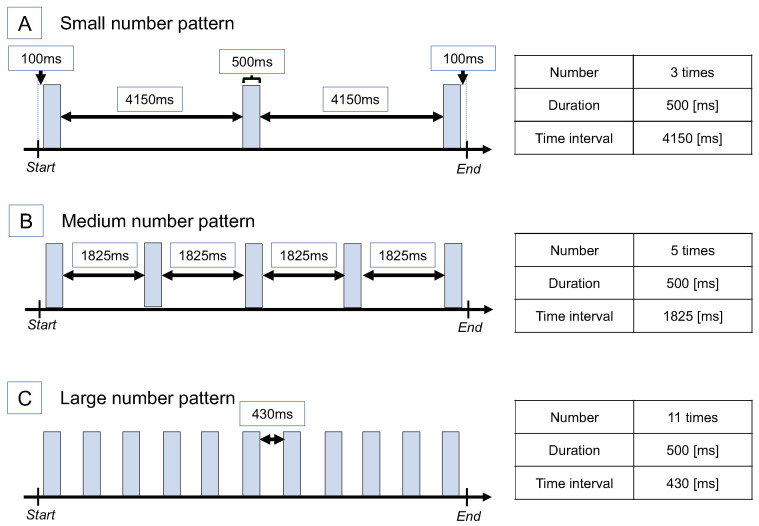
Stimulus pattern of Experiment 1. Three patterns with different numbers of stimuli. Reprinted/adapted with permission from Ref. [11] 2021, ACM.

We prepared three stimulus patterns with different numbers of stimuli for 10 s.Figure 4 shows the designed stimulus patterns. The first one, “Small number pattern”, has three stimuli. The second one, “Medium number pattern”, has five stimuli. The third one, “Large number pattern”, has eleven stimuli. The time interval between the stimuli was set due to the following reasons so that the whole time was 10 s, the start point of the first tactile stimulus was 100 ms after the start of the time, the endpoint of the last tactile stimulus was 100 ms before the end of the time, and the duration of the tactile stimulus was 500 ms.

The reasons for setting up these patterns of stimuli are the following. Firstly, to examine the tendency of the change of the subjective time with the change of the number of stimuli, we thought it was enough to prepare three stages of stimuli. Next, the number of stimuli was set to an odd number to make the time interval between stimuli equal and unify the beginning and end of the stimuli in all patterns. In addition, we set an odd number since the previous study [14] using tactile stimuli reported that the illusion was maximized with an odd number when the time interval between stimuli was evenly spaced. In this way, the minimum number of stimuli was set to three. Then, the stimulus duration was set to 500 ms since it was easy to perceive the stimuli. The maximum number of stimuli was set to 11 to keep the time interval without stimuli at about 500 ms. In addition, since the previous study [1] reported no change in the subjective time when the number of auditory stimuli was continuous (e.g., 12 and 11), the condition of the continuous number of stimuli was omitted.

### 5.2. Experimental Procedure

The experimental task was to compare the subjective time of two different stimulus patterns with a different number of stimuli. Subjects experienced the two stimulus patterns and answered which one was longer or shorter. This task is called “Paired comparison” in previous studies and was adopted based on the previous study [1,14,15,52,53].

The experimental procedure was as follows. Subjects were seated wearing the wrist-worn device. In one trial of the task, the subjective times of the two stimulus patterns were compared. One trial consisted of three parts: 10 s to perceive the first stimulus pattern, 3 s to rest, and 10 s to perceive the second stimulus pattern. The subjects were not informed that the duration of all stimuli was 10 s. The information at the beginning and end of the stimulus presentation was presented in visual text (start, finished) and chime sounds from a laptop computer. Subjects experienced the two types of stimuli and answered the former is longer, the latter is longer, or both are the same. This trial was conducted for a total of three pairs, in which 2 patterns were paired from 3 types of stimulus patterns. Since the perceptual order of the two stimuli to compare might influence the subject’s results in this task, the same pair was verified twice by switching the order of the perception of the two types of stimuli to compare, and the average of the two trials was used as the subject’s score. The perceptual order of the two types of stimuli to compare was randomized. The execution order of the three pairs was randomized. It took 5 min to explain and prepare the experiment and 3 min for the task.

### 5.3. Result

**Figure 5:**
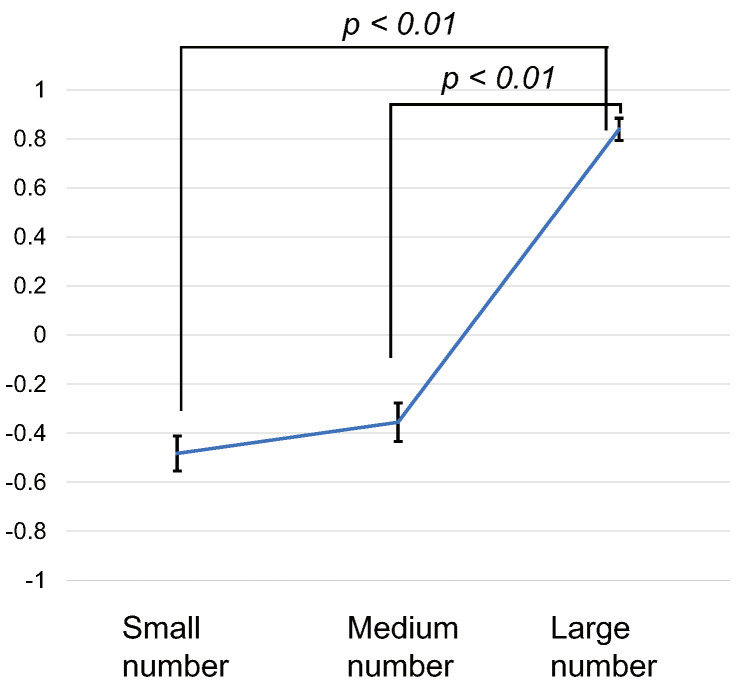
Results of Experiment 1. Scores for each stimulus pattern.

We calculated the score for each stimulus pattern as follows. A score of 1 was given to the stimulus pattern answered as long, a score of −1 to the stimulus pattern answered as short, and a score of 0 to the stimulus pattern answered as the same. The mean of the scores for each stimulus pattern was calculated for each individual. Next, the statistical analysis was a within-subjects design ANOVA and a multiple comparison test using the Bonferroni method. The average scores of all subjects for each stimulus pattern are shown in Figure 5. The error bar is the standard error. The higher the score, the longer the subjective time was perceived to be in the stimulus pattern. The ANOVA showed a significant difference between conditions (F(2, 58)=78.95, p<0.01, effectsize=1.650). Multiple comparison tests showed the following results. The Large number pattern was significantly longer than the Small number pattern (p<0.01) and Medium number pattern (p<0.01). The larger the number of times of stimuli, the longer the score was.

### 5.4. Discussion

The results showed that the number of tactile stimuli affected the subjective time. Specifically, the subjective time increased with increasing the number of stimuli, and the subjective time decreased with decreasing the number of stimuli. This tendency was the same as the filled-duration illusion and the previous study using tactile stimuli [14]. The previous study [14] used 1 s of stimuli and stimuli to the index finger of the hand. On the other hand, we showed that the subjective time can be manipulated with 10 s of stimulation and the stimuli of the wrist-worn device. This result showed the feasibility of our proposed method and useful findings that the number of tactile stimuli of the wrist-worn device can affect the subjective time.

The results also showed that the effect of the number of stimuli on the subjective time was different for each perceptual channel. This result of our study using tactile stimuli was opposite the result of the previous study. The previous study using auditory stimuli [1] reported that the larger the number of stimuli used, the shorter the subjective time became. If we present the user with a stimulus that causes a trend opposite the intended change in the subjective time, the user’s experience may be impaired. Therefore, it is necessary to verify the effect of stimuli on the subjective time for each perception. The findings of this study can be useful for selecting appropriate tactile stimuli in subjective time manipulation. For example, when a short subjective time is desirable, it is necessary to reduce the number of tactile stimuli.

## 6. Experiment 2

Although Experiment 1 evaluated how the change in the number of stimuli affected the subjective time, the duration of one stimulus was fixed. Therefore, in this experiment, we evaluated whether the subjective time changes in response to the change in the duration of the tactile stimuli. The number of subjects was 30, and they were the same as the ones in Experiment 1.

### 6.1. Stimuli

**Figure 6:**
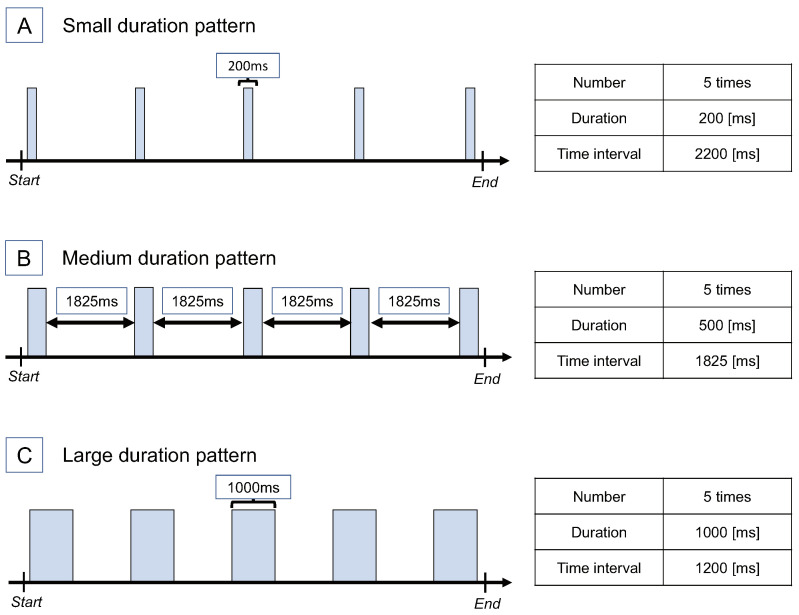
Stimulus pattern of Experiment 2. Three patterns with a different duration of stimuli.

We prepared three stimulus patterns that had different durations of the stimuli, although the number of stimuli in 10 s was the same. Specifically, the designed stimuli are shown in Figure 6. The first one, “Small duration pattern”, had a duration of the stimulus of 200 ms. The second one, “Medium duration pattern”, had a duration of the stimulus of 500 ms. The third one, “Large duration pattern”, has a duration of the stimulus of 1000 ms. The duration of the “Small duration pattern” was set to 200 ms because it was considered to be difficult to perceive a duration of less than 200 ms. The other setting was determined due to the following reasons that the whole time was 10 s, the start point of the first tactile stimulus was 100 ms after the start of the time, the endpoint of the last tactile stimulus was 100 ms before the end of the time, and the number of tactile stimuli was five.

### 6.2. Experimental Procedure

The experimental task and procedure were the same as Experiment 1. One trial of the task was to compare the subjective time of two stimulus patterns. This trial was conducted for a total of three pairs, in which 2 patterns were paired from 3 types of stimulus patterns. The rest of the procedure was the same as Experiment 1.

### 6.3. Result

**Figure 7:**
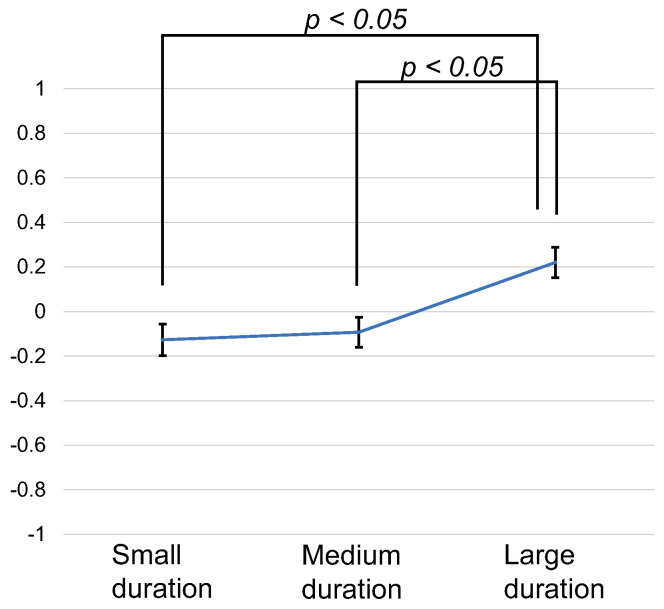
Results of Experiment 2. Scores for each stimulus pattern.

The same scoring and statistical analysis as Experiment 1 were performed. The average scores of all subjects for each stimulus pattern are shown in Figure 7. The error bar is the standard error. The ANOVA showed a significant difference between conditions (F(2, 58)=4.86, p<0.05, effectsize=0.409). Multiple comparison tests showed the following results. There was a significant difference between the stimulus patterns of the Small duration pattern and Large duration pattern (p<0.05). There was a significant difference between the stimulus patterns of the Medium duration pattern and Large duration pattern (p<0.05). The larger duration of the stimuli, the higher the score was.

### 6.4. Discussion

The results showed that the subjective time changed according to the change in the duration of the tactile stimuli. Specifically, there was a tendency that a longer duration of the stimuli changed the subjective time to longer and a lesser duration of the stimuli changed the subjective time to shorter. The effect of the duration of the stimuli on the subjective time was also consistent with our hypothesis based on the filled-duration illusion. This result showed that the change in the subjective time can be manipulated by changing the duration of the tactile stimuli, although it is less clear than the change in the number of stimuli in Experiment 1. We considered that a stimulus pattern with a wider difference in duration compared to this experiment would have a stronger effect, although it was difficult to make further duration differences with a time setting of 10 s.

On the other hand, the change in the subjective time due to the change in this factor of the duration of stimuli has not been verified in previous studies using any perceptual stimuli such as visual, auditory, and tactile. Therefore, the results of the change in the subjective time caused by this factor will provide new insight into the filled-duration illusion.

## 7. Experiment 3

Although Experiments 1 and 2 focused on the number and duration of the stimuli, the time interval between stimuli was fixed. Therefore, in this experiment, we evaluated whether the subjective time changes in response to the change in the time interval of stimuli. The number of subjects was 30, and they were the same as the ones in Experiment 1.

### 7.1. Stimuli

**Figure 8:**
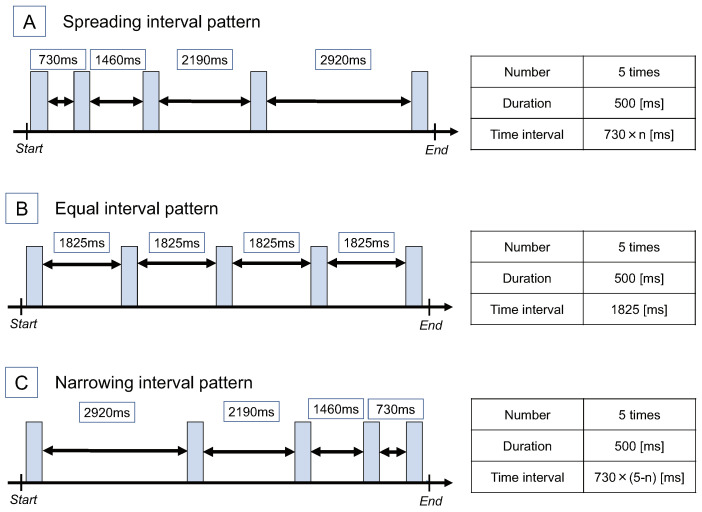
Stimulus pattern of Experiment 3. Three patterns with different time intervals of stimuli.

We prepared three stimulus patterns that had different time intervals of stimuli, although the number and duration of stimuli in 10 s were the same. Specifically, the designed stimuli are shown in Figure 8. The first one, “Spreading interval pattern”, had a time interval of the stimulus of (730×n) ms (*n* is an integer of 0<n<5). The second one, “Equal interval pattern”, had a duration of the stimulus of 1825 ms. The third one, “Narrowing interval pattern”, had a time interval of the stimulus of 730×(5−n) ms (*n* is an integer of 0<n<5). The number and duration of all stimulus patterns were set to 5 and 500 ms, respectively.

### 7.2. Experimental Procedure

The experimental task and procedure were the same as Experiment 1. One trial of the task was to compare the subjective time of two stimulus patterns. This trial was conducted for a total of three pairs, in which 2 patterns were paired from 3 types of stimulus patterns. The rest of the procedure was the same as Experiment 1.

### 7.3. Result

**Figure 9:**
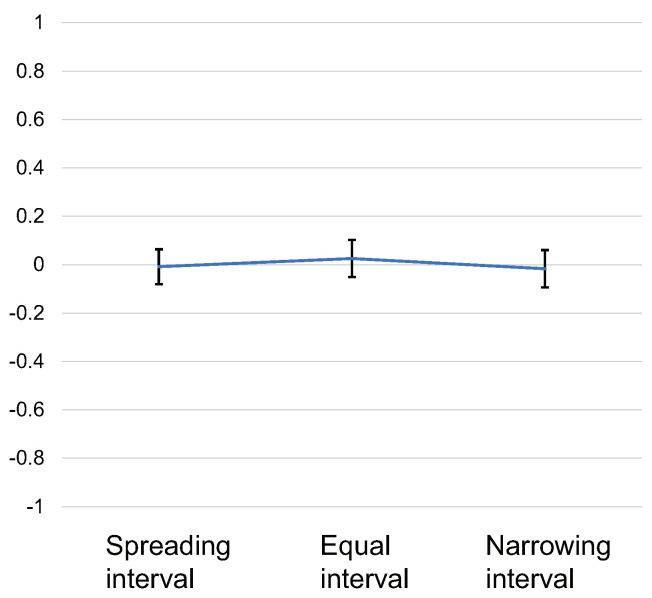
Results of Experiment 3. Scores for each stimulus pattern.

The same scoring and statistical analysis as in Experiments 1 and 2 were performed. The average scores of all subjects for each stimulus pattern are shown in Figure 9. The error bar is the standard error. The ANOVA results showed no significant differences between conditions (F(2, 58)=0.09, p=n.s, effectsize=0.056). Multiple comparison tests also showed no significant differences.

### 7.4. Discussion

It was found that the subjective time did not change in a constant trend even when the factor of the time interval changed. On the other hand, this result showed that the effect of the time interval of stimuli on the subjective time differed among the perceptual channels. The results of the previous study [1], which used auditory stimuli, showed that the subjective time changed by the change in this factor of the time interval, which was different from the results of our study. This result showed that it is necessary to clarify the phenomena occurring in each perceptual channel when investigating the effects of perceptual stimuli on the subjective time, as in Experiment 1. There are two possible reasons why auditory sensation was affected, but tactile sensation was not. The first reason is that tactile sensation may not be affected by the change in the time interval of this stimulus. The second reason is that auditory sensation may be more sensitive to changes in the time interval than tactile sensation. Therefore, it is possible that a more pronounced time interval of the tactile stimuli than this experiment could affect the subjects.

## 8. Experiment 4

In this experiment, we evaluated how much the tactile stimuli can change the subjective time with respect to the objective time. The number of subjects was 30, and they were the same as the ones in Experiment 1.

### 8.1. Stimuli

We prepared six stimulus patterns from Experiments 1, 2, and 3. The six stimulus patterns were “Small number pattern” and “Large number pattern” in Experiment 1, “Small duration pattern” and “Large duration pattern” in Experiment 2, and “Spreading interval pattern” and “Narrowing interval pattern” in Experiment 3.

### 8.2. Experimental Procedure

The experimental task was to experience 10 s with the stimuli and answer how many seconds they felt like. This task is called verbal estimation in previous studies. This task was adopted based on the previous study [1,18,54]. The experimental procedure was as follows. Subjects were in a sitting position with the wrist-worn device attached to their arm. One trial of the task was answering the subjective time of one stimulus pattern. One trial consisted of three parts: 10 s without stimulus, 3 s of rest, and 10 s with stimulus. Subjects answered with a number for the subjective time of the stimulated period compared to the no-stimulus period. Subjects were simply instructed to respond with a number, and no decimal point was specified. Each subject could respond with any number, including decimals, such as 9.5 s. The cues for the start and end of the stimulus presentation were the information of “start” and “stop”, which were presented by visual texts and sounds from a laptop. This task was performed for six different stimulus patterns. The subjects were informed that the no-stimulus period was 10 s and were not informed that the stimulated period was also 10 s. The subjects understood the objective time of 10 s before the task of each stimulus because they experienced 10 s without the stimulus just before experiencing 10 s of each stimulus. The order of stimulus patterns was randomized. The experience of 10 s without the stimuli was performed in all trials. It took 5 min to explain the experiment and wear the wristband and 6 min for the task.

### 8.3. Result

**Figure 10:**
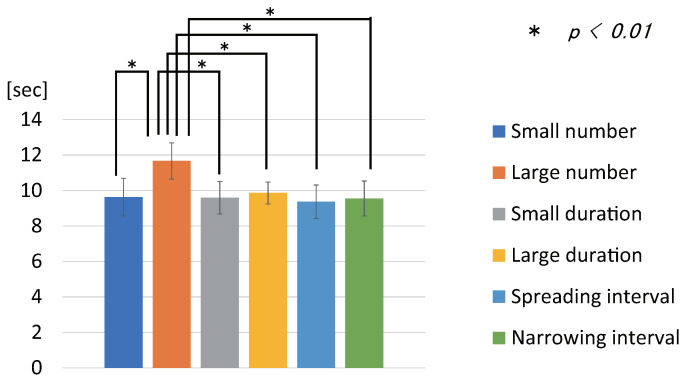
Results of Experiment 4. Scores for each stimulus pattern.

The statistical analysis was a within-subjects design ANOVA and a multiple comparison test using the Bonferroni method.Figure 10 shows the mean of the seconds of the subjective time for each stimulus pattern. The error bar is the standard error. Table 1 shows the degree of the change in the subjective time for each stimulus pattern. The ANOVA showed a significant difference between conditions (F(5, 145)=35.16, p<0.01, effectsize=1.101). Multiple comparison tests showed the following results. There was a significant difference between the stimulus patterns of the Large number pattern and the other patterns (p<0.01). Multiple comparison tests showed the following results. The “Small number pattern” made the subjective time 9.63 s, which was 3.7% shorter than the actual time. The “Large number pattern” made the subjective time 11.67 s, which was 16.7% longer than the actual time. The “Small duration pattern” made the subjective time 9.60 s, which was 4% shorter than the actual time. The “Large duration pattern” made the subjective time 9.87 s, which was 1.3% shorter than the actual time. The “Spreading interval pattern” made the subjective time 9.37 s, which was 6.3% shorter than the actual time. The “Narrowing interval pattern” made the subjective time 9.55 s, which was 4.5% shorter than the actual time. The results indicated that the stimuli implemented in our study could make the subjective time feel shorter or longer than the actual time.

### 8.4. Discussion

The experimental results showed that the subjective time changed compared to the actual time. The subjective time for the five stimulus patterns was shorter than the actual time of 10 s. The shortest subjective time was about 9.37 s, which was 6.3% shorter than the actual time. These results showed that tactile stimuli can shorten the subjective time with respect to the actual time. In addition, the results, in which five stimulus patterns were felt for less than 10 s, suggest that the subjective time is often reduced by the presence of tactile stimuli rather than their absence. Note that there were no significant differences among the five stimulus patterns that were shorter than 10 s, so it is not possible to say which one felt the shortest. Next, the longest subjective time was about 11.67 s, which was 16.7% longer than the actual time. This is thought to be because the change in the number of stimuli was very perceptible and effective. From the experimental results, it was confirmed that the tactile stimuli implemented in our study can make the subjective time feel shorter or longer than the actual time. The degree of the change in the subjective time was about 23% (from −6% to +17%).

## 9. General Discussion, Limitations, and Future work

### 9.1. Degree of the Change in the Subjective Time by Tactile Stimulation

Our proposed method can shorten the subjective time. In detail, 10 s became 6.3% shorter than the actual time. The previous study using auditory stimuli showed that the subjective time was shortened by 7.6% [1]. Therefore, we consider that the effect of our method using tactile stimuli is almost all the same as the effect of the previous study. On the other hand, the effect of our method using tactile stimuli was assumed to be weaker than the effect of the visual progress bar. This assumption was based on the previous study [19,20], wherein the subjective time was shortened by 11% with the visual progress bar, although the experimental conditions of the previous study were not the same as those of our study. These results indicate that stimuli of different perceptual channels can cause different degrees of the effect on the subjective time. However, the appropriate situation is different for the stimuli of each perceptual channel, such as tactile stimuli, auditory stimuli, and visual stimuli. Therefore, we consider that users can use tactile stimuli to complement the shortcomings of methods using auditory stimuli and visual stimuli.

Our proposed method can lengthen the subjective time. In detail, 10 s became about 17% longer than the actual time. There are few studies that have examined stimuli patterns that lengthen the subjective time. Therefore, we consider that stimuli patterns that lengthen the subjective time are valuable. Such stimuli patterns can be useful where users want to increase the subjective time, such as fun time and rest time.

We consider that our study provided new findings not found in the previous study using tactile stimuli [14]. Firstly, the previous study using tactile stimuli showed that the subjective time of 1 s was changed. On the contrary, our study showed that the subjective time of 10 s was changed. Secondly, in the previous study, the tactile stimulation of a stationary device was presented to the index finger of the right hand. On the contrary, in our study, the tactile stimulation of the wrist-worn device was created and presented to the wrist. Thirdly, the previous study conducted only the task of paired comparison, which is similar to Experiment 1 in our study. On the contrary, our study was conducted such that the task of paired comparison and Experiment 4 showed the rate of change of the subjective time with respect to the objective time. Thus, the previous study’s purpose is not a proposal of technology to control subjective time. We consider that our study provided new findings and confirmed the feasibility of the technology to control the subjective time using the tactile stimuli of the wearable device.

### 9.2. Applicability of Our Results

In our study, we clarified both the phenomenon that subjective time changes with the perception of tactile stimuli from information devices and the manipulation method. The results indicate the following two possibilities, which have implications for the design of many information devices capable of presenting tactile stimuli.

First, this study showed that a subjective time manipulation method is possible in the presentation of tactile stimuli by information devices. The results of this experiment using tactile stimuli from a wrist-worn device can be directly applied to a smartwatch. This method can also be applied to existing wearable devices, mobile devices, and stationary devices if they are equipped with vibration elements. Furthermore, the vibrating element can be easily attached to devices and tools other than computers. The method could be replicated through accessories that we wear every day (clothes, shoes, belts, necklaces, etc.) or other objects (chairs, desks, etc.).

Secondly, when presenting tactile stimuli with information devices, it is necessary and possible to consider changes in subjective time. As mentioned in Section 1, subjective time may affect the user experience. If we design tactile stimuli for information devices without considering the impact on subjective time, we may inadvertently damage the user experience. On the other hand, the results of this research are expected to prevent such problems and to be useful for the design of information devices and applications that take the phenomenon into account.

The application scenarios are as follows. First, there are scenarios where the waiting time of smartwatch applications can be shortened, for example the time to launch and install applications and the time to measure mental and physical states (e.g., blood oxygen level or stress) in healthcare applications. There are also scenarios to shorten the waiting time that occurs when a smartwatch is linked to a mobile and ubiquitous device, for example the time to receive and visualize updates (game content, GPS fixes, etc.) needed when using games or weather forecasting applications and the time to display Instream ads, which is boring when using video or music streaming services (Youtube, Spotify, etc.). There are other scenarios that can shorten the time of real-world events. For example, the subjective time of pain caused by a short medical procedure (e.g., injection) can be shortened. An example of exercise is high-intensity interval training (HIIT), which is a repetition of high-intensity exercise and rest in tens of seconds. When performing HIIT, it is possible to shorten the duration of high-intensity exercise while lengthening the rest period, which can feel long.

In addition, we showed that there are new stimulus factors that affect the subjective time. To the best of our knowledge, few studies have investigated the change in the subjective time that is caused by the change in the duration of a stimulus. Our study confirmed that the subjective time was changed by changing this factor. This result can contribute to the field of research on illusions that change the subjective time.

### 9.3. Limitations and Future Works

We have several future works: (1) Young Asians mainly participated in this experiment, and the effectiveness of the proposed method was not verified on various attributes of people. Therefore, we plan to conduct experiments with various attributes of people in the future. (2) Since our proposed method was applied to the wrist-worn device in this study, we plan to verify whether various wearablesand information devices can use the proposed method. (3) We also plan to improve the effectiveness of our method, such as implementing a method that uses multiple perceptual channels of tactile stimuli, auditory stimuli, and visual stimuli. (4) Although these experiments were conducted in an environment like a quiet waiting period, the same as the previous study, we plan to verify whether our approach is effective in an environment with a variety of noises. We believe that our approach is effective as long as users can recognize the stimuli presented to them. (5) All tactile stimuli were of sufficient intensity to be recognizable to the user and not painful. Although this paper assumed that the intensity of the tactile stimuli does not affect the degree of the effect of our approach as long as stimuli are recognizable, we plan to verify changes in the subjective time between different stimulus intensities. (7) If the number of stimuli is changed, the time interval between stimuli will also change. When using a different number of stimuli compared with this experiment, it is recommended to examine the effects of the different number of stimuli based on the same process as in this experiment. For example, we conducted Experiments 2 and 3 with the number of stimuli fixed at 5 and should investigate what effects occur when the number of stimuli is set to a number other than 5. Note that we assumed that similar trend effects would occur even if the number of stimuli were different from those in the our experiment.

## 10. Conclusions

In this study, we proposed a method to manipulate the subjective time by using tactile stimuli from a wearable device. Our method used wrist-worn devices and can be used at any time without blocking visual or auditory perception channels. We implemented a wrist-worn tactile stimulus presentation device and three types of tactile stimuli presentation methods. We conducted four experiments. In Experiment 1, we evaluated the effects on the subjective time that were caused by the number of tactile stimuli within the same time period. The results showed that the subjective time became longer as the number of stimuli increased. In Experiment 2, we evaluated the effects on the subjective time that were caused by the duration of tactile stimuli. The results showed that the subjective time became longer as the duration of stimuli increased. In Experiment 3, we evaluated the effects on the subjective time that were caused by the time intervals of the tactile stimuli. The results showed that the manipulation of this factor did not change the subjective time in a constant trend. In Experiment 4, we selected the six stimuli used in each of the three experiments and evaluated the degree to which these stimuli changed the subjective time with respect to the objective time. All evaluation results clarified the elements of the tactile stimuli that intentionally changed the subjective time and confirmed that our method can change the subjective time by about 23% (from −6% to 17%). While most previous studies that manipulate subjective time using information devices have focused on visual and auditory stimuli, few studies have focused on tactile stimuli. Therefore, we believe that our study greatly contributes to the design of information devices and the improvement of user experience.

## Figures and Tables

**Table 1:** Results of Experiment 4. Degree of change in the subjective time for each stimulus pattern.

Stimulus Pattern	The Subjective Time (s)	Degree of the Change
Small number pattern	9.63 (SD = 1.07)	−3.7%
Large number pattern	11.67 (SD = 1.02)	16.7%
Small duration pattern	9.60 (SD = 0.92)	−4%
Large duration pattern	9.87 (SD = 0.62)	−1.3%
Spreading interval pattern	9.37 (SD = 0.95)	−6.3%
Narrowing interval pattern	9.55 (SD = 0.99)	−4.5%
